# Complementary Metaproteomic Approaches to Assess the Bacterioplankton Response toward a Phytoplankton Spring Bloom in the Southern North Sea

**DOI:** 10.3389/fmicb.2017.00442

**Published:** 2017-03-24

**Authors:** Lars Wöhlbrand, Bernd Wemheuer, Christoph Feenders, Hanna S. Ruppersberg, Christina Hinrichs, Bernd Blasius, Rolf Daniel, Ralf Rabus

**Affiliations:** ^1^General and Molecular Microbiology, Institute for Chemistry and Biology of the Marine Environment (ICBM), Carl von Ossietzky University of OldenburgOldenburg, Germany; ^2^Genomic and Applied Microbiology and Göttingen Genomics Laboratory, Institute of Microbiology and Genetics, Georg-August-University GöttingenGöttingen, Germany; ^3^Mathematical Modelling, Institute for Chemistry and Biology of the Marine Environment (ICBM), Carl von Ossietzky University of OldenburgOldenburg, Germany

**Keywords:** metaproteomics, bacterioplankton, algal bloom, sample preparation, mass spectrometry

## Abstract

Annually recurring phytoplankton spring blooms are characteristic of temperate coastal shelf seas. During these blooms, environmental conditions, including nutrient availability, differ considerably from non-bloom conditions, affecting the entire ecosystem including the bacterioplankton. Accordingly, the emerging ecological niches during bloom transition are occupied by different bacterial populations, with *Roseobacter* RCA cluster and SAR92 clade members exhibiting high metabolic activity during bloom events. In this study, the functional response of the ambient bacterial community toward a *Phaeocystis globosa* bloom in the southern North Sea was studied using metaproteomic approaches. In contrast to other metaproteomic studies of marine bacterial communities, this is the first study comparing two different cell lysis and protein preparation methods [using trifluoroethanol (TFE) and in-solution digest as well as bead beating and SDS-based solubilization and in-gel digest (BB GeLC)]. In addition, two different mass spectrometric techniques (ESI-iontrap MS and MALDI-TOF MS) were used for peptide analysis. A total of 585 different proteins were identified, 296 of which were only detected using the TFE and 191 by the BB GeLC method, demonstrating the complementarity of these sample preparation methods. Furthermore, 158 proteins of the TFE cell lysis samples were exclusively detected by ESI-iontrap MS while 105 were only detected using MALDI-TOF MS, underpinning the value of using two different ionization and mass analysis methods. Notably, 12% of the detected proteins represent predicted integral membrane proteins, including the difficult to detect rhodopsin, indicating a considerable coverage of membrane proteins by this approach. This comprehensive approach verified previous metaproteomic studies of marine bacterioplankton, e.g., detection of many transport-related proteins (17% of the detected proteins). In addition, new insights into e.g., carbon and nitrogen metabolism were obtained. For instance, the C1 pathway was more prominent outside the bloom and different strategies for glucose metabolism seem to be applied under the studied conditions. Furthermore, a higher number of nitrogen assimilating proteins were present under non-bloom conditions, reflecting the competition for this limited macro nutrient under oligotrophic conditions. Overall, application of different sample preparation techniques as well as MS methods facilitated a more holistic picture of the marine bacterioplankton response to changing environmental conditions.

## Introduction

Cultivation-independent analysis of marine bacterioplankton communities targeting 16S rRNA as well as environmental DNA and RNA applying next-generation sequencing techniques greatly advanced our understanding of their diversity and ecology (e.g., Venter et al., 2004; Frias-Lopez et al., 2008; Gifford et al., 2011; Vila-Costa et al., 2012). While metagenomic studies have provided insights into the metabolic potential of the communities, metatranscriptomic analysis generated functional data allowing first hints on microbial activity. In addition, *in situ* activity may be assessed by metaproteomics, analyzing the proteins, i.e., the catalytically active molecules, formed by the community in a given habitat (for overview see Hettich et al., 2012; Abraham et al., 2014). Metaproteomics has been successfully applied to diverse habitats ranging from low-complexity acid mine drainage biofilm (e.g., Ram et al., 2005), activated sludge (e.g., Wilmes and Bond, 2004), human microbiome (e.g., Chen et al., 2008) to the ocean (e.g., Giovannoni et al., 2005; Sowell et al., 2009; Morris et al., 2010; Teeling et al., 2012).

During phytoplankton blooms, large amounts of organic matter are generated by primary production (Arrigo, 2005; Bunse and Pinhassi, 2017). Marine bacteria play an important role in the decomposition of this organic matter, since they remineralize > 50% during and after bloom events (Cole et al., 1988; Kerner and Herndl, 1992; Ducklow et al., 1993). However, diverse environmental factors are influenced by the bloom, including limitation of nutrient availability for the marine bacterioplankton. Therefore, understanding the complex dynamics and interactions between bacterial communities and phytoplankton blooms is essential to assess the ecological impact of bloom events.

Annually recurring phytoplankton spring blooms can be observed in the North Sea, representing a typical coastal shelf sea of the temperate zone. Especially its southern region, the German Bight, is highly productive due to the continuous nutrient supply by rivers (McQuatters-Gollop et al., 2007; Wiltshire et al., 2008, 2010). A dynamic succession of distinct bacterial clades before, during, and after bloom events in the North Sea was observed in recent studies (Alderkamp et al., 2006; Alonso and Pernthaler, 2006a,b; Teeling et al., 2012). They indicate that specialized bacterial populations occupy transitory ecological niches provided by phytoplankton-derived substrates. Metagenomic, -transcriptomic and -proteomic analysis of the diversity and activity of marine bacterioplankton during the same bloom event in the North Sea (Heligoland) showed that members of the *Rhodobacteraceae* and SAR92 clade exhibited high metabolic activity levels (Teeling et al., 2012; Klindworth et al., 2014). In two previous studies, structural and functional differences of the free-living bacterioplankton community in response to a *Phaeocystis globosa* bloom in the southern North Sea in spring 2010 were investigated using comparative metagenomic and metatranscriptomic approaches (Wemheuer et al., 2014, 2015). It was shown that the phytoplankton spring bloom significantly affected bacterioplankton community structures and the abundance of certain bacterial groups, e.g., significantly higher abundance of the *Roseobacter* RCA cluster and the SAR92 clade during a bloom. In addition, functional differences were investigated by comparative metagenomic and metatranscriptomic approaches revealing differences in bacterial gene expression inside and outside of the investigated bloom.

Metaproteomic analysis of environmental samples in particular is challenged by the sample’s inherent high organismic diversity, coupled to the complexity and wide abundance range of the protein complement of each organism. While the latter demands highly accurate peptide separation and mass spectrometric analysis, the preceding cell lysis and subsequent sample processing determine the protein share that is accessible to analysis. Especially in the case of environmental prokaryotic samples, a high diversity of cell wall structures is encountered, ranging from “standard” Gram-positives/-negatives to highly specialized structures, e.g., in case of Planctomycetes (Fuerst, 2005). This diversity necessitates methods for cell lysis that cover all members of the prokaryotic community. Detergent-based methods for protein preparation allow for efficient cell lysis and protein solubilization. However, detergents have to be depleted (e.g., chaotropes as urea) or completely removed (e.g., strong ionic detergents as SDS) from the sample prior to proteolytic digest and subsequent MS analysis to allow for efficient proteolysis and mass acquisition. In previous studies, only a single method for cell lysis and protein preparation was applied to analyze the metaproteome of marine bacterioplankton communities (e.g., Sowell et al., 2009; Morris et al., 2010; Teeling et al., 2012).

In this study, two different types of cell lysis and subsequent protein/peptide separation were applied to analyze the protein complement of the bacterioplankton community within and outside a *P. globosa* dominated algal bloom in the southern North Sea: (i) chemical lysis applying TFE coupled to in-solution digest and MS analysis as well as (ii) mechanical lysis using bead beating (BB) coupled to SDS-PAGE pre-fractionation, in-gel digest and MS analysis. In addition, two different mass spectrometric techniques were applied to analyze nanoLC-separation peptides of the TFE-lysed samples: (i) online by ESI-iontrap MS and (ii) offline by MALDI-TOF MS. Final protein identification was based on a corresponding metagenome/-transcriptome derived protein database. This methodological spectrum was applied to extend our understanding of microbial adaptation to nutrient limitation in the marine habitat.

## Materials and Methods

### Sampling

**FIGURE 1:**
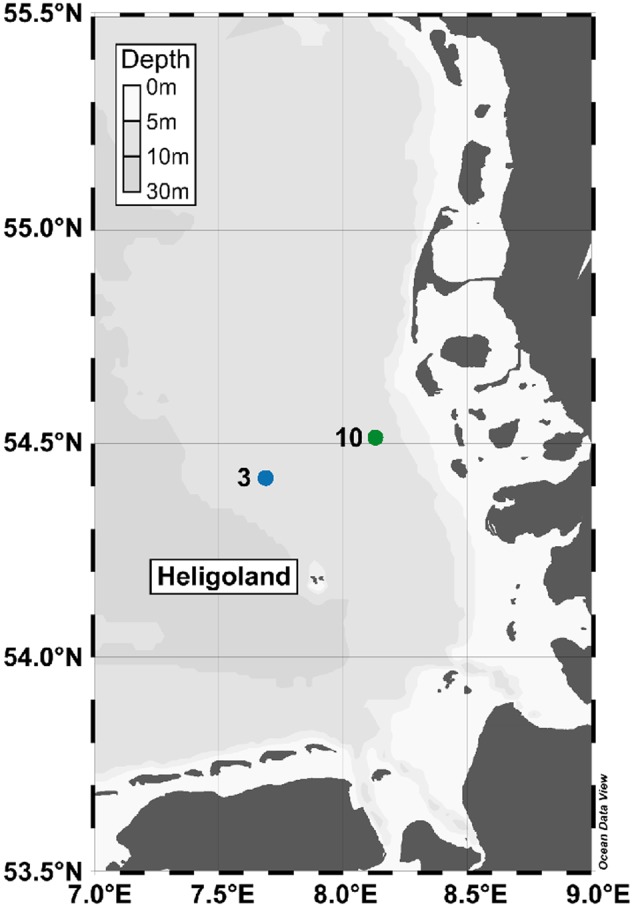
**Map of the German Bight indicating the sampling stations 3 (outside bloom) and 10 (inside bloom) sampled in May 2010.** Shading of the water masses reflects seafloor depth below sea surface. The map was generated using Ocean Data View software package (version 4.7.6; Schlitzer R. 2016; http://odv.awi.de).

Water samples were collected in the southern North Sea at 13 different stations in May 2010 on board RV Heinke (HE327) to investigate bacterial community composition (Wemheuer et al., 2014). At two of these stations additional samples were collected inside (station 10) and outside (station 3) an algal bloom (2 m water depth) for this study (**Figure 1**). Water from eight CTD-mounted 5 L Niskin bottles was pooled in an ethanol-rinsed PE barrel and sequentially filtered through a 10 μm prefilter prior to a sandwich of a precombusted (4 h at 450°C) glassfiber filter (47 mm diameter, Whatman GF/D; Whatman, Maidstone, UK) and a 3.0 μm polycarbonate filter (47 mm diameter, Nuclepore; Whatman). One liter of the obtained filtrate was further filtered through a sandwich of a precombusted (4 h at 450°C) glassfiber filter (47 mm diameter, Whatman GF/F; Whatman) and a 0.2 μm polycarbonate filter (47 mm diameter, Nuclepore, Whatman). Filters were frozen in liquid nitrogen and stored at -80°C until usage.

The presence of an algal bloom, as indicated by satellite data, was confirmed by determination of chlorophyll a and phaeopigments as described in detail by Wemheuer et al. (2014). In addition, suspended particular matter (SPM), particulate organic carbon (POC), particulate organic nitrogen (PON), dissolved inorganic nutrients (nitrate, nitrite, and phosphate) and bacterioplankton cell numbers were determined as described Wemheuer et al. (2014).

### Processing and Analysis of Metagenomic and –Transcriptomic Datasets to Generate a Protein Database for Identification

Metagenomic and metatranscriptomic data sets used in this study were obtained from Wemheuer et al. (2015). Following data processing and assembly, all obtained contigs were joined and open reading frames (ORFs) were predicted for all contigs using Prodigal version 2.6 (Hyatt et al., 2010). Obtained protein sequences were clustered at 80% sequence similarity employing Usearch version 8.0.1623 (Edgar, 2010). During clustering, short peptide sequences (< 50 aa) were removed. Obtained protein sequences were functionally classified using UProC version 1.2 (Meinicke, 2015). Corresponding nucleotide sequences were taxonomically classified using kraken (Wood and Salzberg, 2014).

To determine which ORFs were expressed, metatranscriptomic datasets were mapped on the assembled contigs using Bowtie 2 version 2.2.4 (Langmead and Salzberg, 2012) with one mismatch in the seed and multiple hits reporting enabled. Ribosomal RNA was removed from metatranscriptomic datasets prior to mapping employing SortMeRNA version 2.0 (Kopylova et al., 2012). The number of unique sequences per gene was calculated as described previously (Wemheuer et al., 2015). In addition, coverage was determined for the rRNA-depleted datasets using nonpareil version 2.4 (Rodriguez and Konstantinidis, 2014). Corresponding curves were subsequently generated in R (version 3.2.5; R Development Core Team 2014^1^).

### Protein Extraction

Protein extraction was performed either by direct cell lysis on the filter or by means of BB. In case of direct cell lysis (adapted from Wang et al., 2005), one eighth of a filter sandwich was cut, 200 μL of 50% (v/v) TFE in 100 mM ammonium bicarbonate added and incubated for 5 min in an ultrasound bath. Disulfide bonds were reduced with 10 mM DTT by incubation for 45 min at 55°C followed by alkylation with 50 mM iodoacetamide for 30 min at room temperature in the dark. Subsequently, the suspension was centrifuged for 30 min at 20,000 *g* and 20°C followed by pooling the supernatant of two filter eighths. After addition of an equal amount of HPLC-grade water, a five-fold excess of ice-cold acetone was added and the solution was incubated over night at -20°C. Precipitated proteins were collected by centrifugation (5,700 *g*, 1 h at 4°C; Rotanta RP, Hettich, Beverly, MA, USA) and the obtained pellet was washed twice with 80% v/v ice-cold acetone. The pellet was air dried and resuspended in digestion buffer (50 mM Tris-HCl pH 8.0, 1 M urea). One microgram of trypsin (Trypsin GOLD, Promega, Mannheim, Germany) was added for proteolytic digest and incubated at 37°C over night. Aliquots were frozen in liquid nitrogen and stored at -80°C until use. Three half filters were prepared per station.

The second extraction method was modified from the one described by Teeling et al. (2012). Half a 47 mm filter was cut into small pieces and transferred into a BB tube containing 1.15 g 1.0 mm silica and 0.65 g 0.5 mm yttria-stabilized zirconium oxide spheres (MP Biomedical, Santa Ana, CA, USA). Following addition of 700 μL lysis buffer (50 mM Tris-HCl pH 8.0, 2% w/v SDS, 10% v/v glycerol, 0.1 M DTT) BB was performed for 15 s at a speed of 10 m/s (FastPrep-24 5G; MP Biomedical) with subsequent 5 min incubation on ice and repeated thrice. The suspension was incubated 10 min at 95°C. Following centrifugation (10 min, 20,000 *g*, 4°C) the supernatant was transferred into ultracentrifuge tubes and ultra-centrifuged for 1 h at 100,000 *g* and 4°C (Ultima Max XP equipped with the MLA130 rotor; Beckman Coulter, Krefeld, Germany). Proteins of the supernatant were precipitated by addition of five volumes ice-cold acetone, incubation at -20°C over night and centrifugation for 2 h at 5,700 *g* and 4°C. The obtained pellet was washed with 80% v/v ice cold acetone twice and the pellet air dried prior to resuspension in sample buffer (50 mM Tris-HCl pH 8.0, 10% w/v SDS, 10% v/v glycerol, 0.1 M DTT). The entire protein preparation was subjected to separation by SDS-PAGE (12% acrylamide) using mini gels (7 cm long, 0.75 mm thick) and the Mini-PROTEAN Tetra System (Bio-Rad Laboratories, Munich, Germany). The PageRuler Unstained Protein Ladder (ThermoFisher Scientific, Rockford, IL, USA) was used as molecular size marker. Following electrophoresis, gels were stained with colloidal Coomassie Brilliant Blue according to the method described by Neuhoff et al. (1988). Three half filters were prepared per station.

### Peptide Analysis by nanoLC ESI-Iontrap MS/MS

Aliquots (15 μL) of the in-solution digested samples (TFE) were separated by nanoLC (Ultimate3000 nanoRSLC; ThermoFisher Scientific, Germering, Germany) operated in trap column mode (3 μm beads, 75 μm inner diameter, 2 cm length; ThermoFisher Scientific) equipped with a 25 cm separation column (2 μm beads, 75 μm inner diameter, ThermoFisher Scientific) and applying a linear 240 min gradient from 2% v/v to 50% v/v acetonitrile with subsequent re-equilibration (eluent A: 0.1% v/v formic acid; eluent B: 80% v/v acetonitrile, 0.1% v/v formic acid). The nanoLC effluent was continuously analyzed by an online-coupled iontrap mass spectrometer (amaZon speed ETD; Bruker Daltonik GmbH, Bremen, Germany) using an electrospray ion source (Captivespray; Bruker Daltonik GmbH) operated in positive ion mode as described in detail before (Wöhlbrand et al., 2016).

In case of SDS-PAGE separated samples, the entire sample lane was cut into eight pieces, which were further cut into small pieces of ∼1–2 mm^2^ and subjected to in-gel digest as described by Kossmehl et al. (2013). Generated peptides were analyzed by nanoLC ESI-iontrap MS/MS (setup see above) using a linear 130 min gradient from 2% v/v to 50% v/v acetonitrile with subsequent re-equilibration.

### Peptide Analysis by nanoLC MALDI-TOF/TOF MS/MS

Peptide samples obtained from the direct extraction, i.e., in-solution digest (TFE), were additionally separated by nanoLC (setup see above) coupled to a fraction collection robot (Proteineer FC II; Bruker Daltonik GmbH). The latter continuously mixed the nanoLC eluent with a matrix solution (consisting of 22% v/v of a saturated α-cyano-4-hydroxycinnamic acid solution in 90% v/v acetonitrile, 0.1% v/v trifluoroacetic acid, and 0.1% v/v ammonium-phosphate as well as 0.1% v/v trifluoroacetic acid in 95% v/v acetonitrile) and spotted onto a 384-spot anchorchip target (Bruker Daltonik GmbH) at a flow rate of 0.3 μL/min. Peptides were separated applying a linear gradient of 120 min and fraction collection was performed from 20 to 84 min of this gradient. The peptide standard II mixture (Bruker Daltonik GmbH) was manually applied to calibration spots used for internal mass calibration. Prepared targets were analyzed by a MALDI-TOF/TOF mass spectrometer (ultrafleXtreme; Bruker Daltonik GmbH) using the WARP-LC software (version 1.3; Bruker Daltonik GmbH). Initially, MS analysis was performed for all prepared spots. Subsequently, masses of interest for MS/MS analysis were determined for the entire target that fulfilled the following criteria: signal-to-noise ≥ 20, minimum mass distance to co-eluting compound 1.0 Da, compound mass tolerance 15 ppm. Masses present in more than 60% of all fractions were regarded as background and not considered. MS/MS of masses of interest were acquired for spots revealing the most intense MS signal. Following MS/MS measurement, all MS and MS/MS spectra were combined into a single file containing all compounds and transferred to ProteinScape for protein identification (see below).

### Protein Identification

Protein identification was performed via the ProteinScape platform (version 3.1; Bruker Daltonik GmbH) on an in house Mascot server (version 2.3; Matrix Science Ltd., London, UK) against the generated metagenome-/-transcriptome-based database and applying a target-decoy strategy. Mascot search criteria were as follows: enzyme, trypsin; fixed modification, carbamidomethylation (C); variable modification, oxidation (M); mass values, monoisotopic; max missed cleavages, 1; significance threshold, *p* < 0.05; instrument type, ESI-TRAP or MALDI-TOF-TOF, respectively. Assessment of the Mascot search result was performed accepting only peptides with a Mascot score ≥ 25.0, minimum peptide length of 5 and a false discovery rate < 1.0%. In case of ESI-iontrap, the mass tolerance was set to 0.3 Da (MS) and 0.4 Da (MS/MS) and for MALDI-TOF to 100 ppm (MS) and 0.87 Da (MS/MS). Peptides were identified by at least two spectra meeting the identification criteria (on average 3.6).

In case of measured technical replicates (i.e., same sample preparation), respective peptides were compiled using the protein extractor implemented in ProteinScape. Similarly, peptides of all eight gel slices per sample were compiled yielding a single list of proteins per MS-technique and station sample preparation. A comparative table of all identified proteins with detailed identification information is provided as **Supplementary Table S1**. Protein functions were assessed by functional domains (Pfam) present in the metagenome-derived primary sequence (for details see section on metagenome generation). The mass spectrometry proteomics data have been deposited to the ProteomeXchange Consortium via the PRIDE (Vizcaíno et al., 2016) partner repository with the dataset identifier PXD004944.

### Assessing Similarity of Samples (Multidimensional Scaling)

Sample dissimilarity was calculated according to the Bray–Curtis dissimilarity (Bray and Curtis, 1957) based on presence or absence (coded as 1 or 0, respectively) of each of the 585 assigned different proteins (similar to Kossmehl et al., 2013). The dissimilarity matrix of all pairwise distances was subsequently visualized using multidimensional scaling (MDS; Kruskal and Wish, 1978; Zech et al., 2011). Positions in the 2D plane were scanned for local minima with the Markov chain Monte Carlo sampling method parallel tempering (Swendsen and Wang, 1986; Geyer, 1991), and results were controlled both visually (Shepard diagrams) and through Kruskal’s stress formula 1 (Kruskal, 1964). Differences between sample groups were tested for significance by permutational MANOVA (PERMANOVA) (Anderson, 2001), based on protein presence-absence.

## Results and Discussion

### Characteristics of Analyzed Samples

In this study, the proteomic response of the pelagic bacterial community toward a phytoplankton bloom in the southern North Sea was analyzed. Samples were taken outside (station 3) and inside (station 10) a *Phaeocystis globosa* dominated algal bloom at 2 m water depth (**Figure 1** and **Table 1**). The microalgae *P. globosa* is globally distributed and its blooms have been observed in many marine environments such as the coast of the eastern English Channel, the southern North Sea and the south coast of China (Schoemann et al., 2005). Moreover, it is considered to be responsible for harmful algal blooms (Veldhuis and Wassmann, 2005). While the salinity of both stations was similar (31.4 and 31.1 psu at non-bloom and bloom, respectively), temperature was slightly higher (9.4°C vs. 11.4°C) and the bacterial abundance decreased inside the bloom (2.57 × 10^6^ vs. 1.21 × 10^6^ cells/mL). In addition, the chlorophyll a and phaeopigment contents were > 6-fold higher within the bloom (1.12 vs. 6.93 μg/L and 0.25 vs. 2.10 μg/L, respectively). Overall, most of the determined environmental parameters (**Table 1**) were significantly linked to the presence of an algal bloom, as outlined in detail by Wemheuer et al. (2014).

**Table 1:** Position, time and environmental parameters at the sampling sites and of the retrieved samples.

	Non-bloom (station 3)	Bloom (station 10)
Date of sampling	05/26/2010	05/28/2010
Latitude (°N)	54.4223	54.5135
Longitude (°E)	7.6833	8.128
Water depth (m)	2.0	2.0
Temperature (°C)	9.43	11.40
Salinity (psu)	31.42	31.11
Fluorescence (FU)	0.20	2.27
Transmission (%)	87.98	74.79
Density (g/L)	1024.27	1023.70
Chlorophyll a (μg/L)	1.12	6.93
Phaeopigments (μg/L)	0.25	2.10
Phaeopigments (% total Chl)	18.3	23.3
Bacterial abundance (10^6^/mL)	2.57	1.21
Suspended particulate matter (mg/L)	4.60	7.50
Particulate organic carbon (μg/L)	291.2	737.5
Particulate organic nitrogen (μg/L)	43.0	95.3
Nitrate (μM)	7.4	3.7
Nitrite (μM)	0.25	0.29
Mononitrogen oxides (μM)	7.65	3.98
Phosphate (μM)	0.020	0.070

*Significance of differences between the bloom and non-bloom conditions of all sites of the sampling campaign were tested by either the Student’s two-sample *t*-test (homogeneous variances) or the Welsh Two Samples *t*-test (heterogenous variances) for normally distributed samples and with the Wilcoxon–Mann–Whitney test for non-normally distributed samples (for details see Wemheuer et al., 2014).*

### The Metagenome-Based Protein Database

**FIGURE 2:**
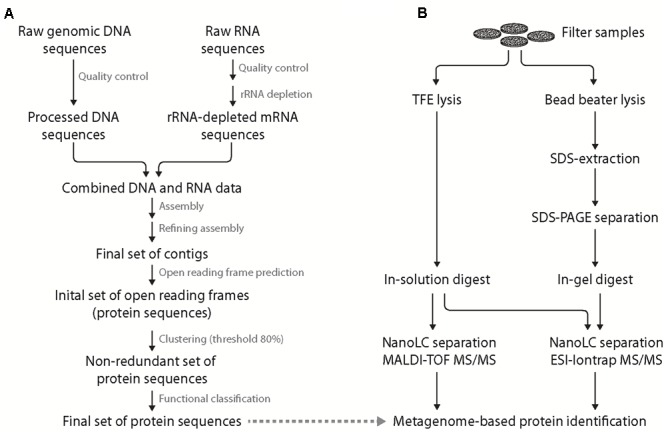
**Integrated scheme of a “meta” protein sequence database generation and complementary metaproteomic approaches.** Generation of metagenome-/-transcriptome-based, site-specific protein sequence database **(A)**. Initial quality control of the obtained genomic DNA and enriched mRNA (including removal of adaptors and artifacts using Trimmatic) allowed the generation of an initial set of contigs (applying Velvet and 29-109 Kmers). Removal of chimeras and refinement of the assembly yielded the final set of contigs that was used for open reading frame (ORF) prediction. The resulting set of protein sequences was clustered to remove redundant sequences (threshold 80% identity) allowing for unambiguous protein identification. Finally, functional prediction for the non-redundant set of protein sequences was performed. Preparation of proteins from filtered bacterioplankton **(B)** was performed by either trifluoroethanol (TFE) lysis (left) coupled to in-solution digest and MALDI-TOF as well as ESI-iontrap mass spectrometry or bead beater lysis (right) with SDS protein extraction coupled to SDS-PAGE pre-fractionation, in-gel digest and nanoLC ESI-iontrap MS. In both cases, protein identification was based on the generated, site-specific metagenome/-transcriptome database.

After quality filtering, nearly 564 million sequences derived from Illumina sequencing and pyrosequencing were assembled employing velvet and metavelvet at different kmer values (**Figure 2A**). After joining all obtained contigs, a total of 14.6 million contigs with an average insert length of 290 bp were retrieved with 13.8 million being unique across all assemblies. Nearly 6 million ORFs were subsequently predicted from these contigs using prodigal. After removal of redundant sequences, >922,000 proteins with an average length of 111 amino acids were obtained and used as backbone for protein calling. However, only 4% of all protein sequences within the database could be allocated to distinct phyla. The low number of taxonomically assigned reads might be related to the incompleteness of currently available databases or the shortness of some peptide reads as these factors are still very problematic during taxonomic binning approaches (as reviewed by Mande et al., 2012).

To determine the number of proteins found in the meta-transcriptomic and meta-proteomic approaches, rRNA-depleted meta-transcriptomic data for the two sampling stations were mapped on all contigs obtained from the assemblies. Afterwards, the number of sequences per ORF was determined. A total of 239 genes or proteins (40.8%), respectively, were detected by both approaches (**Supplementary Table S1**) illustrating differences between, but also similarities of both approaches.

### Experimental Strategy

In this study, two different methods were applied for cellular lysis and protein extraction of bacterioplankton filter samples (**Figure 2B**). The first method was based on chemical cell lysis using TFE (Wang et al., 2005). The organic solvent TFE in hypotonic aqueous buffer allows for cell lysis on the filter and improves protein solubility and denaturation (Ferro et al., 2000; Chertov et al., 2004). Furthermore, its relatively high volatility allows for in-solution digest, direct nanoLC peptide separation and MS analysis thereby minimizing sample loss during sample preparation. The second method applies mechanical forces (BB) for cell lysis and the ionic detergent SDS for protein solubilization. Subsequent SDS-PAGE pre-fractionation was applied to reduce sample complexity as well as to allow for protease treatment following detergent and salt removal (i.e., in-gel digest; method referred to as BB GeLC in the following). The larger peptide sample volume obtained in the TFE-lysis protocol allowed for peptide analysis by two different MS techniques following nanoLC separation, i.e., online-coupled ESI-iontrap MS and offline MALDI-TOF MS. Application of two MS techniques was performed to assess the influence of different ionization (ESI vs. MALDI) and mass analysis (iontrap vs. TOF) techniques on protein identification in case of the investigated environmental samples.

### Overview of the Proteomic Dataset

Combining all nine replicate analyses per station, a total of 585 different proteins was identified, based on the detection of 1,236 different peptides (**Table 2**). Identified proteins revealed an average Mascot score of 129.2, an average number of 1.9 protein identifying peptides (7.2% of all detected proteins identified by a single peptide) and an average sequence coverage of 23.2% (an overview of all identified proteins per station and method is given in **Supplementary Table S1**). Of all 1,936 detected proteins, about 60% were detected in two or more replicate analyses per station (**Supplementary Figure S1**). Single replicate detection of ∼40% is larger than the previously reported 24% for replicate analysis of the yeast proteome applying 2D LC-MS/MS (Liu et al., 2004). The higher share may be attributed to the large environmental database containing > 900,000 amino acid sequences (yeast 6,600), which makes it more likely that proteins miss the applied strict identification criteria (minimal ion score, 95% significance threshold of Mascot, and the target-decoy-based false discovery rate) and thus are not identified in each replicate. Additionally, detectability of peptides is influenced by technical constraints (e.g., ionization suppression of co-eluting peptides and limited number of MS/MS acquired per full scan MS), which are more likely to occur in case of highly complex samples. The very high complexity of the analyzed environmental samples (covering a wide organismic diversity) thus promotes sporadic protein detection (Hettich et al., 2012).

**Table 2:** Summary of protein identification data.

	Total	ESI BB GeLC	Total TFE	ESI TFE	MALDI TFE
No. identified proteins	1,936	604	1,332	734	598
No. different proteins	585	289	394	289	236
No. identified peptides	3,681	959	2,722	1,429	1,293
Average protein score^a^	129.2	100.6	142.2	127.5	160.3
Average no. peptides^b^	1.9	1.8	2.0	1.9	2.2
Average seq. cov. (%)^c^	23.2	20.5	24.4	23.6	25.4
No. detected MemProt	225	53	173	102	70
No. different MemProt^d^	70	28	55	42	28

*The protein identification parameters are given for the sum of all samples (total) as well as subdivided into the different cell lysis and MS techniques (in case of TFE lysis). BB GeLC, bead beater lysis with subsequent SDS-PAGE fractionation and in-gel digest; TFE, trifluoroethanol lysis with in-solution digest. ^a^Mascot score. ^b^Number of protein identifying peptides. ^c^Seq. cov., sequence coverage. ^d^MemProt, membrane proteins, only different proteins counted.*

**FIGURE 3:**
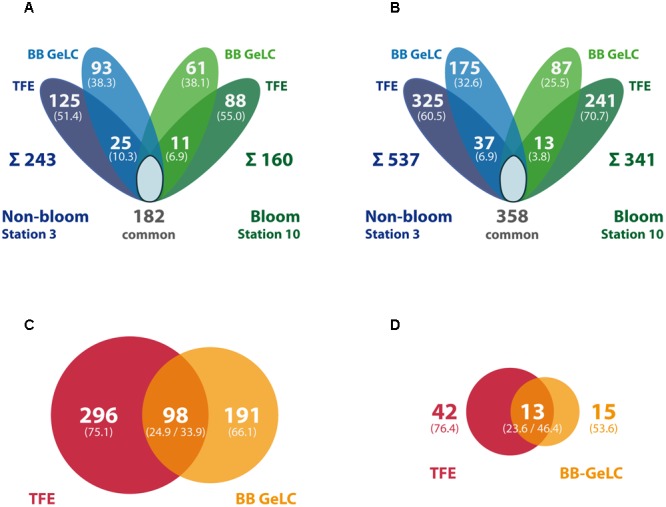
**Summary of identified proteins and peptides per station and preparation method.** The Venn diagrams display the number of proteins **(A)** or peptides **(B)** exclusively detected in samples outside (blue) and inside the bloom (green), respectively, as well as the number of commonly detected proteins (i.e., detected in both stations, including single method detections per station). In addition, station-specific proteins/peptides detected by both, TFE-lysis and bead beater lysis (BB GeLC) are indicated in the overlapping areas. The sum of all station-specific proteins/peptides is given below the diagram. Numbers in brackets give the share of the station-specific proteins or peptides, respectively. The proportion of TFE-lysis (red) vs. BB GeLC (orange) detected proteins is displayed for all detected proteins **(C)** as well as restricted to predicted membrane proteins **(D)**. The number of proteins detected by both methods is given in the overlap region. Areas of the circles **(C,D)** are proportional to the number of proteins represented.

Out of the 585 different proteins, 243 were exclusively detected outside the bloom, while 160 were exclusively detected inside the bloom; 182 proteins were detected at both locations (**Figure 3A**). Correspondingly, more specific peptides were detected outside (537) as compared to inside the bloom (341; 358 peptides present at both stations) (**Figure 3B**). The differences in the number of detected proteins and peptides, respectively, may be attributed to the (i) two-times higher bacterial abundance outside the bloom (**Table 1**) combined with (ii) a higher diversity in the total as well as active microbial community determined by metagenome and -transcriptome analysis (Wemheuer et al., 2015). Notably, 12% of all detected proteins represent predicted integral membrane proteins (**Table 2** and **Supplementary Table S1**) demonstrating coverage of the membrane protein fraction to some extent.

### Significance of Station-Specific Protein Complements

**FIGURE 4:**
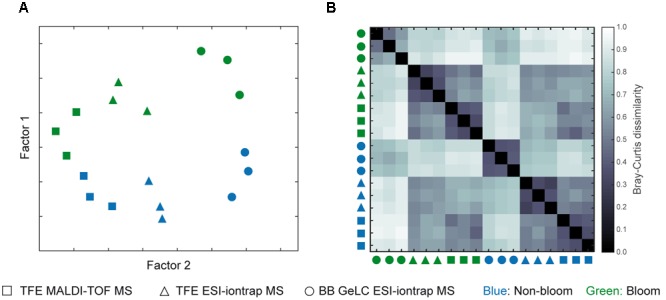
**Overview of differences in generated proteomic data.** Two-factor multidimensional scaling plot **(A)** visualizing differences between all samples included in this study based on the presence or absence of detected proteins. The Bray–Curtis dissimilarity plot **(B)** displays relations of each sample to all others with a value of 0 corresponding to identical samples and 1 to completely different samples (i.e., no common proteins). Non-bloom samples are colored blue while those inside the bloom are green. The symbols refer to the type of cell lysis and protein preparation as well as the MS method applied: square, TFE lysis with MALDI-TOF MS; triangle, TFE lysis ESI-iontrap MS; circle, BB GeLC ESI-iontrap MS.

Identified proteins per station and preparation as well as MS detection method (in case of TFE samples) were tested for significance using a multivariate approach, which considers the protein data (i.e., protein presence or absence) of each station and method replicate (i.e., three replicates each of three distinct methods) as a multidimensional entity. To investigate how the different replicate samples relate to each other, a PERMANOVA (Anderson, 2001) comparing differences within and in-between groups was applied. Here, a group consists of the three replicates per station and measurement technique. In addition, similarities and dissimilarities between each prepared sample were visualized by MDS (**Figure 4A**). A detailed view on the relation (Bray–Curtis dissimilarity) of all samples to each other is provided in **Figure 4B**. Here, a value of 0 corresponds to completely identical samples (i.e., the same proteins were detected in both samples) and 1 to maximally different samples (i.e., samples share no proteins at all).

A clear separation of samples originating from out- and inside the bloom is visualized by the MDS-plot (**Figure 4A**) reflecting the calculated significant difference of the samples (*p* = 0.1), irrespective of the cell lysis and protein preparation method used. Interestingly, replicate groups of the different preparation and measurement techniques of each station revealed clear differences in protein detection (*p* = 0.004). Accordingly, proteins detected among replicates (any combination of station and measurement technique) are highly similar compared to those of other combinations (triplicate to all others < *p* = 0.0005). This tight relationship is also visible in both the MDS- as well as the dissimilarity-plot, where replicate samples are in close proximity and display low Bray–Curtis dissimilarity values, respectively (**Figure 4**). Notably, despite their unique features, TFE lysis samples analyzed by ESI-iontrap or MALDI-TOF MS are more closely related to each other as compared to the BB GeLC treated samples.

### Impact of Protein Preparation and MS Methods on Protein Detection

Distinct sub-proteomes were detected applying the different lysis and protein preparation methods in case of both station samples (**Figure 3A**). Interestingly, the share of the method-specific protein detections of the respective station protein complement is very similar for both stations: 38% and 38% BB GeLC exclusive, 51% and 55% TFE exclusive and only 7 and 10% of the proteins were detected by both methods (values given for out- and inside the bloom, respectively). This reproducible distinctiveness of the detected protein complements with respect to the extraction method but independent of sample origin demonstrates method specific effects on the protein extraction. This effect becomes more evident, considering all identified proteins irrespective of the station: 75% of the proteins detected by the TFE approach were exclusively identified by this method while 66% of the proteins detected by the BB GeLC approach were exclusively identified by this method (**Figure 3C**). Notably, nearly twice as many predicted membrane proteins have been identified applying the TFE method (55) as compared to the BB GeLC method (28) (**Figure 3D**). Hence, a pronounced difference of the TFE and BB GeLC prepared protein complement was shown, demonstrating a high degree of complementarity of these methods. The latter may be attributed to the different chemical attributes of the solvents used. TFE is a small fluoroorganic compound that acts phenol-like in many reactions, though the mechanism of protein solubilization by TFE is not completely understood at present (Kundu and Kishore, 2004). The anionic, micelle forming detergent SDS is a large molecule consisting of a long apolar alkyl chain (C_12_) esterified to sulfuric acid. Protein solubilization is achieved by hydrophobic interaction of its alkyl-moiety with (hydrophobic) amino acids of proteins causing unfolding due to electrostatic repulsion. Based on these chemical differences, the applied methods apparently have (i) a different effectiveness of cell lysis for different bacteria of the sampled community [i.e., in combination with ultrasound (TFE) or bead beating (BB GeLC), respectively] and (ii) a different protein accessibility ultimately affecting solubilization. Efficient protein extraction from environmental samples using TFE was previously reported for an acid mine drainage biofilm sample (Thompson et al., 2008), which, however, exhibits a rather low organismic diversity and comparison to SDS extraction has not been performed. It would be of interest to understand, if different bacterial (or archaeal) phyla are preferentially lysed by either of the methods. This could lead to misinterpretation of obtained metaproteomic data if only one method would be applied. Unfortunately, the very low share of phylogenetically allocable proteins within the present study does not allow for such a survey.

**FIGURE 5:**
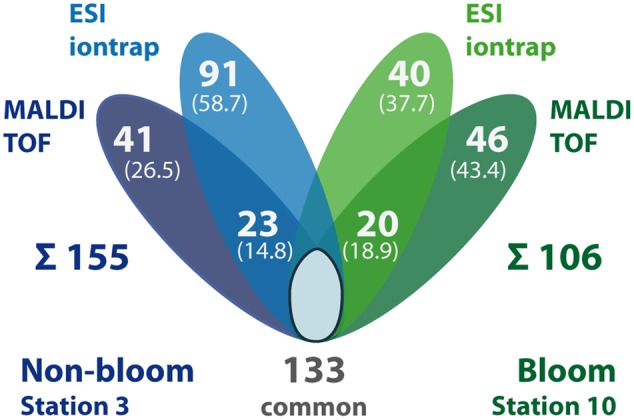
**Summary of identified proteins in TFE-lysis samples per station and MS detection method.** The number of proteins exclusively detected in non-bloom (blue) and bloom samples (green), respectively, as well as the number of commonly detected proteins (i.e., detected in both stations, including single method detections per station) is displayed. In addition, station-specific proteins detected only by ESI-iontrap or MALDI-TOF MS are indicated as well as the number of proteins detected by both MS methods (overlapping areas). The sum of all station-specific proteins is given below the diagram. Numbers in brackets give the share of the station-specific proteins.

The rather large volume of sample obtained by the TFE-lysis protocol allowed for peptide analysis by two different mass spectrometric methods following initial nanoLC separation: (i) online by ESI-iontrap MS and (ii) offline by MALDI-TOF MS. In addition to the different ionization (ESI vs. MALDI) and mass analysis techniques (iontrap vs. TOF), the methods differ with respect to precursor selection for MS/MS fragmentation and fragment analysis (for overview see Wöhlbrand et al., 2013). About 50% of the detected proteins from the TFE-lysis samples were exclusively detected by either one of the two applied MS methods (**Figure 5**). The determined complementarity is even more pronounced than previously reported for the analysis of bovine mitochondrial ribosomes (63% overlap) applying both methods (Bodnar et al., 2003). This proportion is rather similar when only one condition is considered (**Figure 5**) as well as in the case of the predicted membrane proteins (not shown). While proteins detected by both ESI-iontrap MS and MALDI-TOF MS revealed on average two protein identifying peptides and average sequence coverages of ∼24% (23.6% ESI-MS vs. 25.4% MALDI-MS), the average Mascot score of ESI-iontrap MS identified proteins was 127, that of MALDI-TOF MS identified proteins was 160 (**Table 2**), demonstrating the superior mass accuracy of the TOF mass analyzer.

### Bacterioplankton Protein Complements are Affected by the Bloom

**FIGURE 6:**
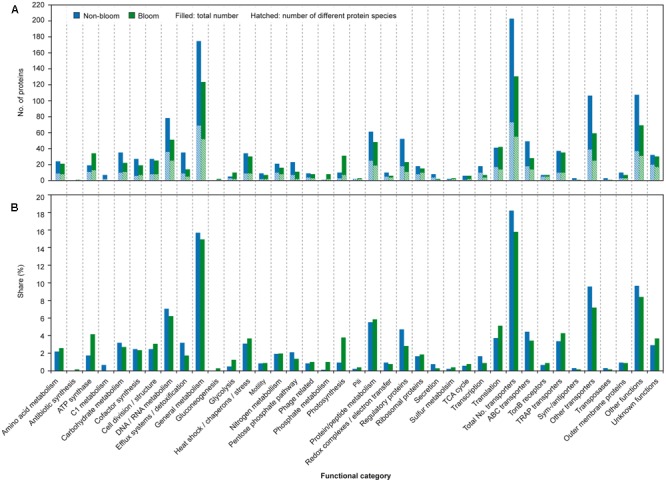
**Overview of proteins according to functional categories.** Indicated is the number of proteins detected **(A)** for non-bloom (blue) and bloom samples (green), respectively. The hatched area refers to the number of detected different proteins. In addition, the share **(B)** of the proteins per category of the non-bloom and bloom proteomes is given.

Identified proteins were categorized according to their predicted functions, revealing that nearly a third of all proteins belong to transporters (17.1%) and proteins of general metabolism (15.3%), respectively (**Figure 6** and **Supplementary Table S2**). Other abundant categories include DNA/RNA (6.7%), protein/peptide (5.6%) or carbohydrate metabolism (3.0%), translation (4.3%), regulatory proteins (3.9%) as well as proteins of unknown functions (3.2%). Although the relative proportion of all categories within the station protein complements is rather similar, the metaproteomic analysis revealed differences between bloom and non-bloom conditions with respect to the number of detected proteins of the different functional categories as well as their relative share of the respective station-proteome.

Outside of the bloom, the number (202 vs. 130) as well as the share of detected transport proteins among the respective station proteome (18.2% vs. 15.8%) is pronouncedly higher as compared to bloom conditions (**Figure 6** and **Supplementary Table S2**). The high share of transport proteins is similar to the SAR11 metaproteome of the Sargasso Sea (17.4%; Sowell et al., 2009) as well as surface waters of the South Atlantic (Morris et al., 2010). The elevated number during non-bloom conditions determined in this study can be ascribed to a 30% higher share of ABC transporter-related proteins (as compared to the bloom conditions). Moreover, a nearly two-fold higher number of different unclassified transport proteins were detected outside the bloom. These unclassified transport proteins comprise a large number of unclassified periplasmic binding proteins that account for 9.5% of the station proteome (**Figure 6** and **Supplementary Table S2**). Furthermore, the larger share of detected proteins involved in protein secretion outside the bloom (0.7% vs. 0.2%) agrees with the higher amount of extracellular binding proteins in this habitat. Overall, the high number of detected ABC-type transport proteins under the non-bloom condition is reminiscent of the situation in the oligotrophic Sargasso Sea (Sowell et al., 2009). Hence, formation of a large number of high affinity transport systems may be beneficial under nutrient limited conditions to scavenge the rare nutrients, a hypothesis previously also raised by Hirsch et al. (1979) and Button (1993). Furthermore, potential involvement of (some) periplasmic binding proteins in signal transduction and chemotaxis (Mowbray and Sandgren, 1998) may facilitate migration toward preferred habitat conditions.

In addition to transport proteins, a higher number and share of regulatory proteins as well as proteins involved in transcription (mainly RNA polymerase subunits) and DNA/RNA metabolism were detected outside the algal bloom (**Figure 6** and **Supplementary Table S2**). The increased abundance of these proteins may represent a state of readiness during the oligotrophic conditions, to quickly respond to nutrients becoming available by promptly forming proteins for their utilization.

Within the bloom, the share of proteins related to photosynthesis was more than two times higher (2.5%) as compared to non-bloom conditions (0.9%) (**Figure 6** and **Supplementary Table S1**) agreeing with higher photosynthetic activity at this station. Notably, these proteins also include a bacteriorhodopsin detected at both stations (TFE lysis combined with MALDI-TOF detection), despite its challenging detectability due to its hydrophobicity and generally low abundance (Stapels et al., 2004). Rhodopsins were previously also detected by membrane targeting proteomics in nutrient-poor surface waters of the southern Atlantic, the productive Benguela upwelling region (Morris et al., 2010) and coastal pacific waters (Giovannoni et al., 2005), suggesting a function not restricted to low-nutrient conditions. Besides photosynthetic proteins, a higher share of ATP synthase proteins (4.1% vs. 1.7%) and proteins involved in translation (5.1% vs. 3.7%) within the bloom may be indicative for a higher metabolic activity of the bacterial community, reflecting the higher nutrient availability. Correspondingly, a higher transcript level of tRNA synthetases within the bloom was previously determined for the same samples (Wemheuer et al., 2015).

Although a similar number of different proteins related to carbohydrate metabolism was detected at both stations (i.e., 11), both identified glycoside hydrolases were detected inside, but only one outside the bloom (**Figure 6** and **Supplementary Table S2**). A higher abundance of such carbohydrate-active enzymes (CAZymes) was previously reported for algal bloom conditions in the North Sea (Teeling et al., 2012) and respective transcripts were detected to be more abundant within the bloom for the same samples as studied here (Wemheuer et al., 2015). The rather low difference between the bloom and non-bloom conditions may be due to the observed increase and subsequent decrease during the succession of the bloom (Teeling et al., 2012). The increased transcript level but rather low difference in protein amount may point to sampling during the increasing phase, so that transcripts may not yet be completely translated into proteins.

Interestingly, different catabolic strategies for glucose seem to be applied under the analyzed conditions. A higher share of proteins involved in the Enter-Douderoff (ED) and pentose phosphate (PP) pathway was present outside the bloom (2.1% vs. 1.3%), while the share of glycolysis proteins was higher within the bloom (0.5% vs. 1.2%) (**Figure 6** and **Supplementary Table S2**). Such increased abundance of ED and PP pathway proteins was previously observed in batch culture for *Phaeobacter inhibens* DSM 17395, a member of the *Roseobacter* group, during growth in minimal medium as compared to complex medium (Zech et al., 2013) agreeing with the *in situ* observation.

Proteins of the C1 pathway were pronouncedly more abundant outside the bloom, comprising 1.0% of the station proteome (0.1% inside the bloom) (**Figure 6** and **Supplementary Table S2**) indicating activity of methylotrophic bacteria. Correspondingly, the active community outside the bloom revealed a pronouncedly higher share of methylotrophic OM43 clade members as compared to bloom conditions (Wemheuer et al., 2015) supporting the metaproteomic finding. Abundant detection of methanol dehydrogenase affiliated to OM43 members was previously also reported for the east Pacific during coastal upwelling (Sowell et al., 2011). Sowell et al. (2011) suggested that methanol, possibly produced by phytoplankton, may represent a significant source of carbon and energy in coastal ecosystems. Detection of C1 pathway related proteins under non-bloom conditions may indicate that also other one carbon compounds, including trimethylamine or dimethylsulfoniopropionate, potentially serve as substrates for methylotrophs in coastal areas.

In accordance with the competition of bacteria and algae for the macro-nutrient phosphate within the algal bloom, proteins involved in phosphate uptake and starvation were more numerous inside the bloom (1.0% vs. 0.1%), which is consistent with previous observations reported by Teeling et al. (2012). The limited amount of nitrogen outside the bloom is reflected by the detection of a higher number of glutamine synthetases (3 out of 4) as well as amidohydrolases (4 out of 5) at this station (only 2 and 1 inside the bloom, respectively) (**Figure 6** and **Supplementary Table S1**). Similarly, proteins involved in nitrogen metabolism, including glutamine synthetase and the nitrogen regulatory protein PII (effecting glutamine synthase activity) were detected in other oligotrophic marine environments (Sowell et al., 2009; Georges et al., 2014). Increased amounts of ammonia assimilating glutamine synthase were reported for diverse bacteria in response to nitrogen limitation (for overview see Leigh and Dodsworth, 2007), which is in accordance with the *in situ* observations of this study.

The similar number of phage-related proteins present in both investigated samples indicates a comparable phage impact under both environmental conditions (**Figure 6** and **Supplementary Table S1**). Detection of station-exclusive phage proteins may be attributed to the differing composition of the community (Wemheuer et al., 2015) which might provoke activity of phages specific for respective phyla.

## Conclusion

This study demonstrates the complementarity and, hence, the value of using two different sample preparation and two different ionization and mass analysis techniques for a comprehensive characterization of environmental samples. Furthermore, a considerable number of membrane proteins were covered without specifically preparing the membrane fraction. This approach revealed that the non-bloom microbial community applies the ED and PP as well as C1 pathway for carbon metabolism and is prepared to adapt to changing conditions as evident from the high share of proteins involved in e.g., high affinity transport, regulation or transcription. In contrast, the bloom community focusses on metabolism of the nutrients present and simultaneously competes with the algae for limited macro-nutrients. Overall, however, the usually limited amount of environmental samples as well as the commonly low cell numbers on the filters restricts repeated analyses accomplishable per sample.

## Author Contributions

LW and RR conceived and designed the experiments; BW performed sampling on BB Heinke and generated the metagenomic/-transcriptomic database; LW, HR, and CH performed the proteomic experiments; LW, CF, and BW analyzed the data; LW, BW, CF, and RR wrote the paper; all authors reviewed, edited and approved the manuscript.

## Conflict of Interest Statement

The authors declare that the research was conducted in the absence of any commercial or financial relationships that could be construed as a potential conflict of interest.

## Footnotes

1 http://www.R-project.org/

## Supplementary Material

FIGURE S1 **Share of replicate protein identifications per station.** Given is the number of repetitive protein identifications per station sample (non-bloom, blue; bloom, green) and their respective share of the station proteome.

TABLE S1 **Summary of all identified proteins**.

TABLE S2 **Functional categorization of identified proteins.** Given is the number and share of identified proteins in total as well as per station (in alphabetical order).

## Abbreviations

BB: bead beating
ESI: electrospray ionization
GeLC: SDS-PAGE decomplexation coupled to in-gel digest and nanoLC separation
MALDI: matrix assisted laser desorption ionization
nanoLC: nano liquid chromatography
SDS: sodium dodecyl sulfate
TFE: trifluoroethanol
TOF: time-of-flight
