# Supplementary figures and images for: Complementary Metaproteomic Approaches to Assess the Bacterioplankton Response toward a Phytoplankton Spring Bloom in the Southern North Sea
Source: Front Microbiol. 2017 Mar 24;8:442. doi: 10.3389/fmicb.2017.00442 (PMC5364173; doi:10.3389/fmicb.2017.00442)

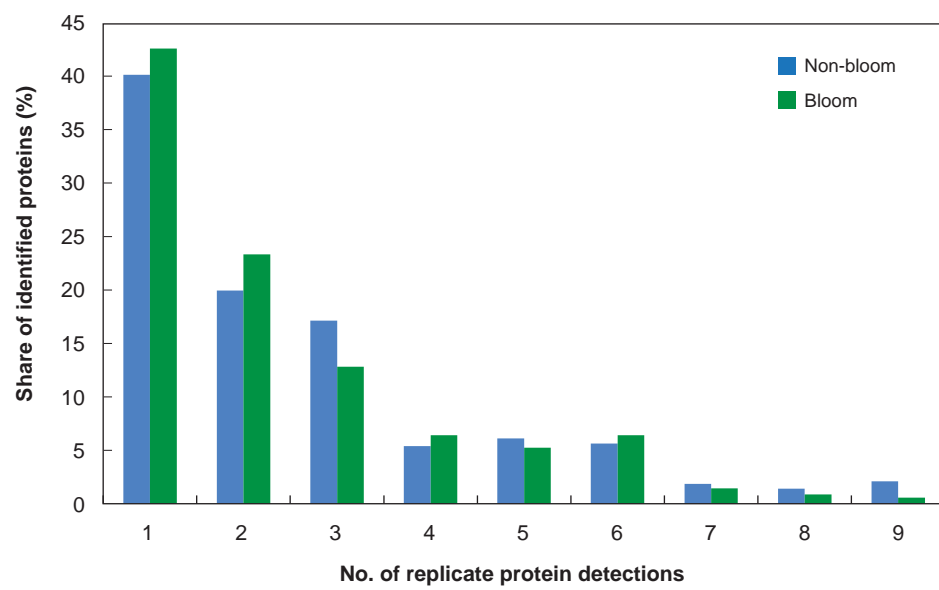

Fig. S1

Supplement: FIGURE S1 — Share of replicate protein identifications per station. Given is the number of repetitive protein identifications per station sample (non-bloom, blue; bloom, green) and their respective share of the station proteome. [file Image_1.pdf]
