# Supplementary material for: Complementary Metaproteomic Approaches to Assess the Bacterioplankton Response toward a Phytoplankton Spring Bloom in the Southern North Sea
Source: Front Microbiol. 2017 Mar 24;8:442. doi: 10.3389/fmicb.2017.00442 (PMC5364173; doi:10.3389/fmicb.2017.00442)
Supplement: TABLE S2 — Functional categorization of identified proteins. Given is the number and share of identified proteins in total as well as per station (in alphabetical order). [file Table_2.docx]

Supplementary Material

**Complementary metaproteomic approaches to assess the bacterioplankton response towards a phytoplankton spring bloom in the southern North Sea**

**Lars Wöhlbrand*, Bernd Wemheuer, Christoph Feenders, Hanna S. Ruppersberg, Christina Hinrichs, Bernd Blasius, Rolf Daniel and Ralf Rabus**

*** Correspondence:** Lars Wöhlbrand: lars.woehlbrand@uni-oldenburg.de

**TABLE S1 | Summary of all identified proteins (provided as separate file)**

**TABLE S2 | Functional categorization of identified proteins**. Given is the number and share of identified proteins in total as well as per station (in alphabetical order).

|  | **Total** | | |  | **Non-bloom** (St. 3) | | |  | **Bloom** (St. 10) | | |  |
| --- | --- | --- | --- | --- | --- | --- | --- | --- | --- | --- | --- | --- |
|  | **Number** | | **Share (%)** |  | **Number** | | **Share (%)** |  | **Number** | | **Share (%)** | |
| **Category** | **all** | **nr^c^** |  |  | **all** | **nr^c^** |  |  | **all** | **nr^c^** |  |  |
| Amino acid metabolism | 45 | 12 | 2.3 |  | 24 | 9 | 2.2 |  | 21 | 8 | 2.5 | |
| Antibiotic synthesis | 1 | 1 | 0.1 |  | 0 | 0 | 0.0 |  | 1 | 1 | 0.1 | |
| ATP sythase | 53 | 20 | 2.7 |  | 19 | 11 | 1.7 |  | 34 | 13 | 4.1 | |
| C1 metabolism | 12 | 3 | 0.6 |  | 11 | 3 | 1.0 |  | 1 | 1 | 0.1 | |
| Carbohydrate metabolism | 57 | 16 | 2.9 |  | 35 | 10 | 3.2 |  | 22 | 11 | 2.7 | |
| CAZymes^a^ | 14 | 2 | 0.7 |  | 9 | 1 | 0.8 |  | 5 | 2 | 0.6 | |
| Cofactor synthesis | 46 | 9 | 2.4 |  | 27 | 6 | 2.4 |  | 19 | 7 | 2.3 | |
| Cell division / structure / envelope | 52 | 12 | 2.7 |  | 27 | 8 | 2.4 |  | 25 | 8 | 3.0 | |
| DNA / RNA metabolism | 129 | 46 | 6.7 |  | 78 | 36 | 7.0 |  | 51 | 25 | 6.2 | |
| Efflux systems / detoxification | 49 | 10 | 2.5 |  | 35 | 9 | 3.2 |  | 14 | 5 | 1.7 | |
| General metabolism | 297 | 96 | 15.3 |  | 174 | 69 | 15.7 |  | 123 | 52 | 14.9 | |
| Gluconeogenesis | 2 | 1 | 0.1 |  | 0 | 0 | 0.0 |  | 2 | 1 | 0.2 | |
| Glycolysis | 15 | 4 | 0.8 |  | 5 | 3 | 0.5 |  | 10 | 2 | 1.2 | |
| Heat shock proteins / chaperons / stress | 64 | 13 | 3.3 |  | 34 | 9 | 3.1 |  | 30 | 9 | 3.6 | |
| Motility | 16 | 3 | 0.8 |  | 9 | 2 | 0.8 |  | 7 | 2 | 0.8 | |
| Nitrogen metabolism | 37 | 15 | 1.9 |  | 21 | 10 | 1.9 |  | 16 | 8 | 1.9 | |
| Pentose phosphate pathway | 34 | 7 | 1.8 |  | 23 | 7 | 2.1 |  | 11 | 2 | 1.3 | |
| Phage related | 17 | 5 | 0.9 |  | 9 | 4 | 0.8 |  | 8 | 3 | 1.0 | |
| Phosphate metabolism | 9 | 3 | 0.5 |  | 1 | 1 | 0.1 |  | 8 | 2 | 1.0 | |
| Photosynthesis | 41 | 8 | 2.1 |  | 10 | 3 | 0.9 |  | 31 | 7 | 2.5 | |
| Pili | 5 | 3 | 0.3 |  | 2 | 2 | 0.2 |  | 3 | 2 | 0.4 | |
| Protein/peptide metabolism | 109 | 31 | 5.6 |  | 61 | 25 | 5.5 |  | 48 | 19 | 5.8 | |
| Redox complexes / electron transfer | 16 | 7 | 0.8 |  | 10 | 5 | 0.9 |  | 6 | 4 | 0.7 | |
| Regulatory proteins | 75 | 23 | 3.9 |  | 52 | 18 | 4.7 |  | 23 | 11 | 2.8 | |
| Ribosomal proteins | 33 | 13 | 1.7 |  | 18 | 8 | 1.6 |  | 15 | 10 | 1.8 | |
| Secretion | 5 | 4 | 0.5 |  | 8 | 4 | 0.7 |  | 2 | 1 | 0.2 | |
| Sulfur metabolism | 5 | 3 | 0.3 |  | 2 | 1 | 0.2 |  | 3 | 2 | 0.4 | |

Continued next page

# TABLE S2 | continued

|  | **Total** | | | | |  | | **Non-bloom** (St. 3) | | | | | |  | **Bloom** (St. 10) | | | | | |  |  |
| --- | --- | --- | --- | --- | --- | --- | --- | --- | --- | --- | --- | --- | --- | --- | --- | --- | --- | --- | --- | --- | --- | --- |
|  | **Number** | | | **Share (%)** | |  | | **Number** | | | | **Share (%)** | |  | **Number** | | | | **Share (%)** | | |  |
| **Category** | **all** | **nr^c^** | |  |  |  | | **all** | | **nr^c^** | |  |  |  | **all** | | **nr^c^** | |  |  |  |  |
| TCA cycle | 12 | 2 | 0.6 | |  | | 6 | | 1 | | 0.5 | |  | | | 6 | | 2 | | 0.7 | | |
| Transcription | 25 | 13 | 1.3 | |  | | 18 | | 10 | | 1.6 | |  | | | 7 | | 4 | | 0.8 | | |
| Translation | 83 | 23 | 4.3 | |  | | 41 | | 17 | | 3.7 | |  | | | 42 | | 14 | | 5.1 | | |
| Transporter^b^ | 332 | 96 | 17.1 | |  | | 202 | | 73 | | 18.2 | |  | | | 130 | | 55 | | 15.8 | | |
| ABC transporter | 77 | 22 | 4.0 | |  | | 49 | | 18 | | 4.4 | |  | | | 28 | | 14 | | 3.4 | | |
| TonB receptors | 14 | 9 | 0.7 | |  | | 7 | | 5 | | 0.6 | |  | | | 7 | | 5 | | 0.8 | | |
| TRAP transporter | 72 | 14 | 3.7 | |  | | 37 | | 10 | | 3.3 | |  | | | 35 | | 10 | | 4.2 | | |
| Sym-/Antiporter | 4 | 2 | 0.2 | |  | | 3 | | 1 | | 0.3 | |  | | | 1 | | 1 | | 0.1 | | |
| Other transporter | 165 | 49 | 8.5 | |  | | 106 | | 39 | | 9.5 | |  | | | 59 | | 25 | | 7.2 | | |
| Transposases | 4 | 1 | 0.2 | |  | | 3 | | 1 | | 0.3 | |  | | | 1 | | 1 | | 0.1 | | |
| Outer membrane proteins | 17 | 4 | 0.9 | |  | | 10 | | 3 | | 0.9 | |  | | | 7 | | 3 | | 0.8 | | |
| Other functions | 180 | 48 | 9.1 | |  | | 107 | | 37 | | 9.6 | |  | | | 69 | | 31 | | 8.4 | | |
| Unknown function | 62 | 31 | 3.2 | |  | | 32 | | 20 | | 2.9 | |  | | | 30 | | 17 | | 3.6 | | |

^a^ Included in carbohydrate metabolism, ^b^Sum of all transport categories listed below; ^c^ non-redundant; only different proteins per category counted
